# Plastic Degradation Potential and Metagenomic Analysis of an Enriched Gut Microbial Consortium from *Tenebrio molitor*

**DOI:** 10.3390/microorganisms14061246

**Published:** 2026-06-02

**Authors:** Qing Qiu, Xuejian Sun, Han Li, Dandan Zhou, Hongliang Huo

**Affiliations:** 1 School of Environment, Northeast Normal University, No. 2555 Jingyue Avenue, Changchun 130117, China; 2 Engineering Research Center of Low-Carbon Treatment and Green Development of Polluted Water in Northeast China, Ministry of Education, Northeast Normal University, Changchun 130117, China

**Keywords:** *Tenebrio molitor*, gut microbiota, metagenomics, degradation genes, degradation pathways

## Abstract

Plastic pollution has become an increasingly severe global environmental issue, highlighting the urgent need for efficient and sustainable biodegradation strategies. In this study, an enriched gut microbial consortium, NE-01 derived from *Tenebrio molitor*, exhibited significant degradation activity toward polystyrene (PS), polyethylene (PE), and polyethylene terephthalate (PET). Metagenomic sequencing revealed that Pseudomonas and Proteobacteria were the dominant taxa, maintaining high community diversity and providing a microbial foundation for the degradation of plastics and other complex organic compounds. Functional annotation and metabolic pathway analysis indicated that xenobiotic biodegradation and metabolism occupied a large proportion of the metabolic network, suggesting the consortium’s potential for degrading exogenous pollutants. Several key genes associated with the degradation of aromatic and halogenated compounds, such as benzoate, toluene, styrene, and bisphenol A, were identified. Metabolic reconstruction further suggested possible degradation pathways for PS, PE, PET, and the plasticizer di(2-ethylhexyl) phthalate (DEHP). This study preliminarily demonstrated that the *T. molitor* gut-derived microbial consortium harbors multiple plastic-degrading genes and provides a theoretical basis for developing green, microbe-based strategies for plastic degradation.

## 1. Introduction

Plastic products have been widely applied in daily life and industrial production due to their excellent mechanical properties, chemical stability, and low cost. However, synthetic polymers such as polystyrene (PS), polyethylene (PE), and polyethylene terephthalate (PET) are extremely difficult to degrade under natural conditions. Their long-term accumulation not only aggravates the pressure of solid waste disposal but also causes serious global ecological problems [1]. A large amount of plastic entering the ocean can entangle aquatic organisms, leading to suffocation and tissue damage, thereby significantly increasing morbidity and mortality rates [2,3]. Meanwhile, plastic debris gradually decomposes into microplastics and nanoplastics, which are widely distributed in soil, aquatic environments, and the atmosphere, and accumulate progressively through food chains, posing potential threats to ecosystem stability and human health [4,5,6,7]. Microplastics have already been detected in human placenta, feces, lung tissue, breast milk, and blood [8], and their long-term exposure risks have raised growing global concern. In addition to the polymers themselves, additives extensively used during plastic production also present significant environmental risks. Among them, phthalate esters (PAEs) are the most common plasticizers. Di(2-ethylhexyl) phthalate (DEHP, also referred to as DHEP) is physically bound to polymer matrices, making it prone to release into the environment during manufacturing and use [9]. Specifically, DEHP acts as a plasticizer that is physically blended rather than covalently bonded with polymer chains, which explains its high mobility and environmental release potential. Previous studies have demonstrated that DEHP exerts endocrine-disrupting effects and potential reproductive and immunotoxicity [10,11], and its persistence and hazardous properties pose considerable challenges for environmental management. Moreover, its primary metabolite, mono(2-ethylhexyl) phthalate (MEHP), has been identified as an even more potent endocrine disruptor, particularly in adipocytes, where it interferes with lipid metabolism and adipogenesis [12]. Therefore, addressing the environmental risks of plastics and their additives simultaneously has become a critical issue in current research.

In recent years, plastic treatment technologies have gradually shifted from incineration and landfilling to photodegradation, thermal degradation, and biodegradation [13,14]. Against this background, microbial degradation has attracted extensive attention due to its green and sustainable nature. Biodegradation utilizes microorganisms and their secreted enzymes to transform recalcitrant plastics into harmless products and is regarded as an important future development direction. Insect gut microbiota, endowed with unique plastic-degrading abilities, have been considered an “emerging resource pool.” Previous studies have demonstrated that insects such as mealworms (*Tenebrio molitor*) [15], superworms (*Zophobas atratus*) [16], Indian mealmoths (*Plodia interpunctella*) [17], and greater wax moths (*Galleria mellonella*) [18,19] possess the capability to degrade multiple polymers. Among them, *T. molitor* exhibits the most remarkable performance, being capable of degrading a wide range of polymers, including PS, LDPE, PP, PVC, PLA, and PET [15,20,21,22,23]. Notably, *T. molitor* larvae can survive on PS or PE as their sole carbon source, with their gut microbiota playing a central role in plastic degradation [24,25]. Several isolates from insect guts, such as *Exiguobacterium* sp. YT2, *Acinetobacter* sp. AnTc-1, and *Klebsiella* sp. EMBL-1, have been verified to efficiently degrade PS and PE [17,26]. Yang et al. also isolated *Bacillus* sp. YP1 and *Enterobacter asburiae* YT1 from the gut of *P. interpunctella* larvae, which exhibited effective PE degradation. Collectively, among diverse insect species, *T. molitor* larvae stand out for their efficiency in degrading various polymers, highlighting their potential as a model system for plastic biodegradation research [15,20,21,22,23].

In particular, little is known about whether insect gut microbiota possess the ability to simultaneously degrade multiple plastics and their additives, which severely limits their potential application in environmental remediation. Previous studies have demonstrated that, compared with single strains, microbial consortia exhibit stronger synergistic potential in the degradation of complex plastic mixtures [27]. In this context, culture-independent omics approaches, such as metagenomics and metatranscriptomics, have rapidly emerged as powerful tools for investigating microbial communities and their functional genes in diverse environments [28]. These approaches overcome the limitations of traditional isolation-based methods and enable the discovery of a broader range of potential plastic-degrading resources [29].

The gut microbiota of *T. molitor* has been reported to possess inherent plastic-degrading potential; however, this process largely depends on the physiological state and survival of the host insect, making it difficult to isolate and utilize functional microorganisms at scale. To overcome this limitation and further explore its biotechnological potential, the gut-derived microorganisms were enriched under in vitro conditions. The enriched microbial consortium exhibited plastic-degrading activity outside the host, providing a foundation for future industrial applications and the development of plastic-degrading enzyme preparations. Therefore, in this study, we enriched the gut microbiota of *T. molitor*, evaluated its degradation performance toward multiple plastics (PS, PE, and PET) and the plasticizer DEHP, and systematically analyzed the potential plastic-degrading genes through metagenomic sequencing and functional annotation. This work provides new insights into the metabolic potential and synergistic mechanisms of insect gut-derived microbial consortia in degrading complex polymer mixtures, offering theoretical support and genetic resources for developing microbe-based strategies for plastic pollution control.

## 2. Materials and Methods

### 2.1. Sample Collection and Plastic Degradation Tests of PS, PE, and PET by Gut Microbiota

#### 2.1.1. Sample Collection and Gut Microbiota Isolation

(1) Rearing and pretreatment of *T. molitor*

Healthy larvae of *Tenebrio molitor* (body length 1.8–2.3 cm) were purchased from Qingyifang Market, Changchun City, Jilin Province, China. The larvae were reared under controlled environmental conditions (25 ± 1 °C, relative humidity 75 ± 5%). To avoid potential interference from residual feed, all larvae were subjected to a 48 h starvation period before the experiment. Subsequently, they were fed with sterilized wheat bran for five consecutive days to restore physiological activity and stabilize the gut microbial community structure.

(2) Aseptic collection of gut samples

On day 5, a subset of larvae was randomly selected for sampling. Prior to dissection, the larvae were surface-sterilized with 75% ethanol for 1 min and rinsed three times with sterile phosphate-buffered saline (PBS) to remove residual ethanol. All subsequent procedures were performed under aseptic conditions: the abdomen was incised with a sterile scalpel, and the intact intestine was carefully excised with sterile forceps and immediately transferred into a sterile centrifuge tube containing autoclaved phosphate-buffered saline (PBS) to maintain bacterial osmotic balance. The intestinal contents were collected, and the resulting suspension was prepared as the source of gut microorganisms.

(3) Obtention of the microorganism consortium

The collected gut contents were inoculated into Luria–Bertani (LB) broth and incubated at 30 °C with shaking at 150 rpm for 24 h to enrich metabolically active gut microorganisms. In this study, we designated the microbial consortium as NE-01. It originated from the intestinal contents of *T. molitor* and represents a subcommunity of gut-derived microorganisms.

(4) Application of the consortium

The NE-01 consortium was subsequently used for plastic degradation experiments and metagenomic sequencing, aiming to investigate its in vitro plastic degradation potential and to identify the key functional genes potentially involved in this process.

#### 2.1.2. Preparation of Plastic Materials

Three representative plastics were selected as substrates for the degradation experiments: polystyrene (PS, –CH_2_–CH(C_6_H_5_)–ₙ–), polyethylene (PE, –CH_2_–CH_2_–ₙ–), and polyethylene terephthalate (PET, –O–CH_2_–CH_2_–O–CO–C_6_H_4_–CO–ₙ–). All plastics were additive-free and purchased from Sigma-Aldrich (St. Louis, MO, USA). Prior to the experiments, the plastic films (thickness approximately 0.1 mm) were sterilized and then added to 100 mL of mineral salt medium (MSM), where each type of plastic served as the sole carbon source in the system.

#### 2.1.3. Plastic Degradation Experiments

For the plastic degradation experiments, the NE-01 microbial suspension cultured in Luria–Bertani (LB) medium for 24 h was centrifuged to collect the cells, followed by three washes with phosphate-buffered saline (PBS). The resulting cell pellets were resuspended in PBS and inoculated into mineral salt medium (MSM) containing polystyrene (PS), polyethylene (PE), or polyethylene terephthalate (PET) as the sole carbon source.

The MSM was prepared by dissolving 2.65 g KH_2_PO_4_, 0.2 g MgSO_4_·7H_2_O, 1.5 g (NH_4_)_2_SO_4_, 4.26 g Na_2_HPO_4_, and 0.02 g CaCl_2_ in ultrapure water. The solution was adjusted to pH 7.0, supplemented with 1 mL of trace element solution, and finally diluted to a total volume of 1000 mL to form the carbon-free basal medium.

The initial cell density of the NE-01 inoculum was adjusted to 1 × 10^8^ CFU/mL, and 5 mL of this suspension was added to 95 mL of mineral salt medium (MSM) to establish a 100 mL degradation system, resulting in an initial bacterial density of approximately 5 × 10^6^ CFU/mL. Plastic materials (PS, PE, or PET) were added at 0.2 g per 100 mL, corresponding to a final concentration of 2 g/L (2000 mg/L). Based on these conditions, the total initial inoculum contained ~5 × 10^8^ CFU, equivalent to approximately 2.5 × 10^6^ CFU per mg of plastic. This quantitative ratio was included to provide a clear reference for evaluating the degradation capacity of the NE-01 consortium.

The cultures were incubated in a shaking incubator at 30 °C and 180 rpm for 30 days to evaluate the degradation of different plastics. Prior to incubation, the plastic films were dried at 30 °C for 24 h and weighed to record their initial mass. After incubation, the PS, PE, and PET films were retrieved and washed with 2% (*w*/*v*) sodium dodecyl sulfate (SDS) solution for 4 h to remove cells attached to the film surface, followed by three rinses with sterile water to ensure cleanliness. The films were then dried at 40 °C for 24 h and weighed using an electronic balance with a precision of 0.00001 g to calculate the plastic weight loss rate.

The degradation rate of plastics was calculated using the following equation:

Degradation rate (%) = [(W_0_ − W_t_/W_0_] × 100%

where W_0_ is the initial dry weight of the plastic film, and W_t_ is the dry weight after incubation.

### 2.2. DNA Extraction of Microbial Samples

Genomic DNA was extracted from the microbial samples using the E.Z.N.A™ Mag-Bind Soil DNA Kit (Omega Bio-tek, Norcross, GA, USA; M5635-02) according to the manufacturer’s instructions. The quality of the extracted DNA was evaluated by agarose gel electrophoresis, and the DNA concentration was measured using the Qubit™ dsDNA HS As-say Kit (Thermo Fisher Scientific, Waltham, MA, USA; Q32854). DNA was further purified with Hieff NGS™ DNA Se-lection Beads (Yeasen Biotechnology, Shanghai, China; 12601ES56), and the concentration of the purified DNA was de-termined with a Qubit 4.0 fluorometer (Thermo Fisher Scientific, Waltham, MA, USA).

### 2.3. Library Construction and Quality Control

DNA libraries were constructed using the Hieff NGS^®^ MaxUp II DNA Library Prep Kit for Illumina^®^ (Yeasen, 12200ES96, San Diego, CA, USA) according to the manufacturer’s protocol. The library preparation workflow included end repair and A-tailing, adapter ligation, bead-based purification, and subsequent library amplification and purification. Library size distribution was verified using 2% agarose gel electrophoresis, and library concentrations were quantified using a Qubit 4.0 fluorometer. The qualified libraries were pooled at equimolar ratios (1:1) for sequencing. Metagenomic sequencing was performed by Sangon Biotech (Shanghai, China) on the Illumina NovaSeq 6000 platform with a paired-end 150 bp (PE150) strategy. Raw reads were processed with FastQC and Trimmomatic for quality control, including adaptor removal, filtering of reads containing >10% ambiguous bases (N), and trimming of low-quality bases (Q < 20). High-quality clean reads were used for downstream analyses. De novo assembly was conducted using MEGAHIT (v1.2.9) based on the De Bruijn graph algorithm. Open reading frames (ORFs) were predicted using Prodigal (v2.6.3), and redundant sequences were clustered using CD-HIT to generate a nonredundant gene catalog. Functional annotation of the gene catalog was performed against the KEGG, COG, and CAZy databases.

### 2.4. Sequencing, Assembly, and Gene Prediction

The pooled libraries were subjected to high-throughput sequencing. Raw reads were processed for quality control to remove adapters and low-quality sequences, yielding clean reads. De novo assembly was performed using assembly tools, followed by the prediction of open reading frames (ORFs) to generate a nonredundant gene catalog (unigenes).

### 2.5. Functional Annotation

Functional annotation of the nonredundant gene catalog was performed following standard metagenomic workflows. Specifically, predicted protein sequences were first aligned against the KEGG database using DIAMOND (v0.8.20) to obtain the corresponding KEGG Orthology (KO) identifiers, which were subsequently mapped to related pathways and modules to elucidate metabolic processes and functional modules. Second, the protein sequences were compared with the eggCOG database using DIAMOND and assigned to functional categories (e.g., metabolism, information processing, and cellular processes) to reveal the distribution of gene functions. Finally, InterProScan or Blast2GO was applied to annotate the protein sequences against the Gene Ontology (GO) database, thereby retrieving functional information in the three major categories: Molecular Function, Cellular Component, and Biological Process.

## 3. Results

### 3.1. Plastic Degradation by the Gut-Derived Microbial Consortium NE-01

**Figure 1:**
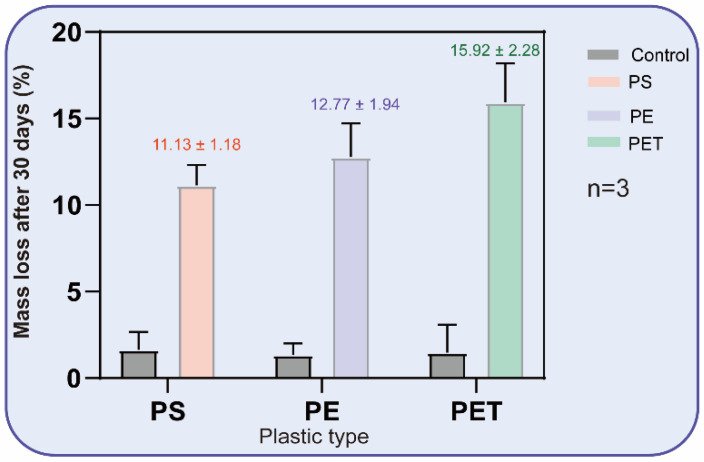
Mass loss after 30 days of PS, PE, and PET degraded by the gut microbial consortium NE-01. Data are presented as mean ± SE (*n* = 3).

The gut-derived microbial consortium isolated from *T. molitor* was designated as NE-01, and its degradation capacity toward three common plastics (PS, PE, and PET) was systematically evaluated (Figure 1). During the 30-day in vitro incubation, NE-01 exhibited clear degradation activity against all three plastics, with mass losses of 11.13% for PS, 12.77% for PE, and as high as 15.92% for PET. These results indicate that NE-01 possesses the potential to degrade multiple types of plastics simultaneously, not only handling conventional polymers such as PS and PE but also efficiently degrading PET. The multifunctional degradation capability of this consortium highlights the application potential of *T. molitor* gut microbiota in plastic biodegradation and provides experimental evidence for the development of efficient and broad-spectrum microbial degradation systems.

### 3.2. Taxonomic Composition of the Gut Microbial Consortium at the Phylum and Genus Levels

**Figure 2:**
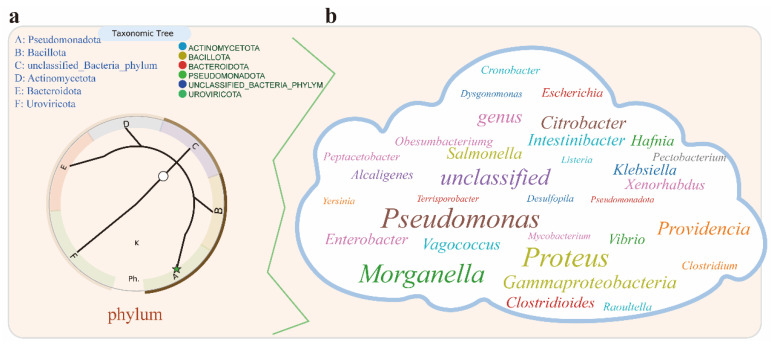
Taxonomic composition of the gut microbiota of *T. molitor* (**a**) phylum level (the letters A–F denote the major taxonomic clades, where A represents Pseudomonadota and B represents Bacillota. The black lines indicate the phylogenetic branches, illustrating the evolutionary relationships among different phyla. The green star marks the primary phylogenetic position of the representative sequence from the NE-01 consortium, which is classified within the phylum Pseudomonadota). (**b**) Genus level.

To further elucidate the molecular basis underlying the plastic degradation capacity of NE-01, metagenomic analysis was performed. Whole-genome sequencing and functional annotation enabled the systematic identification of key enzymes and metabolic pathways associated with plastic degradation, thereby providing molecular-level insights into its degradation mechanisms and theoretical support for the subsequent optimization and application of this consortium. At the phylum level (Figure 2a), the community was mainly composed of Proteobacteria, Firmicutes, and Actinomycetota, with Proteobacteria being the dominant group, indicating its strong adaptability and metabolic advantages in the gut environment. A small proportion of Bacteroidota and other unclassified bacteria were also detected, reflecting the diversity of the community structure. Figure 2b shows the overall composition at the genus level. The analysis revealed that *Pseudomonas* was the most dominant genus (86.06%), markedly higher than other groups, suggesting its potential central role in organic compound degradation. In addition, members of the Enterobacteriaceae family, such as *Morganella*, *Proteus*, and *Providencia*, were also relatively abundant, indicating their importance in nitrogen metabolism and the utilization of complex organic substrates. Meanwhile, other genera including *Hafnia*, *Klebsiella*, *Escherichia*, and *Salmonella* were present at lower abundances but collectively maintained the metabolic diversity of the consortium. Overall, the gut microbial consortium displayed a structural pattern characterized by *Pseudomonas* as the core genus, Proteobacteria as the dominant phylum, and the coexistence of multiple taxa, which provides a solid community basis for the potential degradation of plastics and other complex organic compounds.

### 3.3. Functional Annotation of the Gut Microbiota

**Figure 3:**
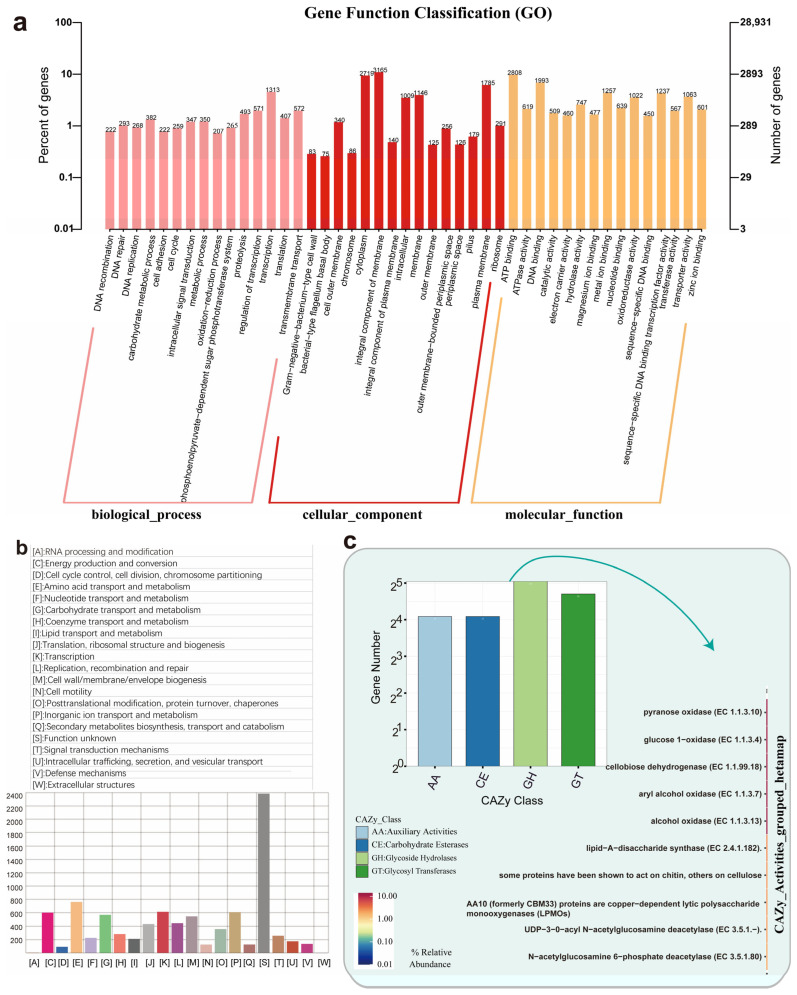
Functional annotation of the gut microbiota (**a**) GO classification; (**b**) COG classification; (**c**) CAZy enzyme family distribution (The curved arrow indicates the high-abundance enzymes annotated from each CAZy family).

Based on the metagenomic annotation of NE-01, functional genes were further systematically analyzed. The GO functional classification (Figure 3a) showed that the annotated genes were mainly enriched in three categories: Biological Process, Cellular Component, and Molecular Function. Within Biological Process, genes involved in metabolism, cellular processes, and response to stimulus accounted for a large proportion; within Cellular Component, genes related to cell structure and cell membranes were abundant; and within Molecular Function, genes associated with catalytic activity and binding activity were most prevalent, suggesting that the gut microbiota has strong potential in material transformation and energy metabolism. The COG functional classification (Figure 3b) further revealed that genes were mainly distributed in amino acid transport and metabolism, energy production and conversion, transcription regulation, and carbohydrate transport and metabolism. Notably, the categories “carbohydrate transport and metabolism” and “energy production and conversion” occupied the largest proportions, indicating that this community is highly adapted to the degradation of organic substrates and energy acquisition. The CAZy family annotation (Figure 3c) showed that multiple classes of carbohydrate-active enzymes were detected in the gut microbiota, including glycoside hydrolases (GHs), glycosyltransferases (GTs), carbohydrate esterases (CEs), and auxiliary activities (AAs), with GHs and GTs being the most abundant, reflecting the strong activity of this community in polysaccharide degradation and glycosyl transfer reactions. In addition, the AA family included multicopper oxidases associated with lignocellulose degradation, such as pyranose oxidase, glucose oxidase, and aryl-alcohol oxidase, suggesting that the gut microbiota of *T. molitor* is not only capable of degrading polysaccharide substrates but also harbors CAZy enzymes related to lignin degradation. The presence of these enzymes reflects the community’s capacity for the metabolism of natural aromatic polymers and provides an enzymatic basis for the degradation of plastics or other recalcitrant aromatic compounds. Collectively, these results revealed that the gut microbiota possesses both efficient carbohydrate metabolic potential and key functions associated with aromatic compound transformation.

### 3.4. KEGG-Based Analysis of Degradation Pathways in the Gut Microbiota

**Figure 4:**
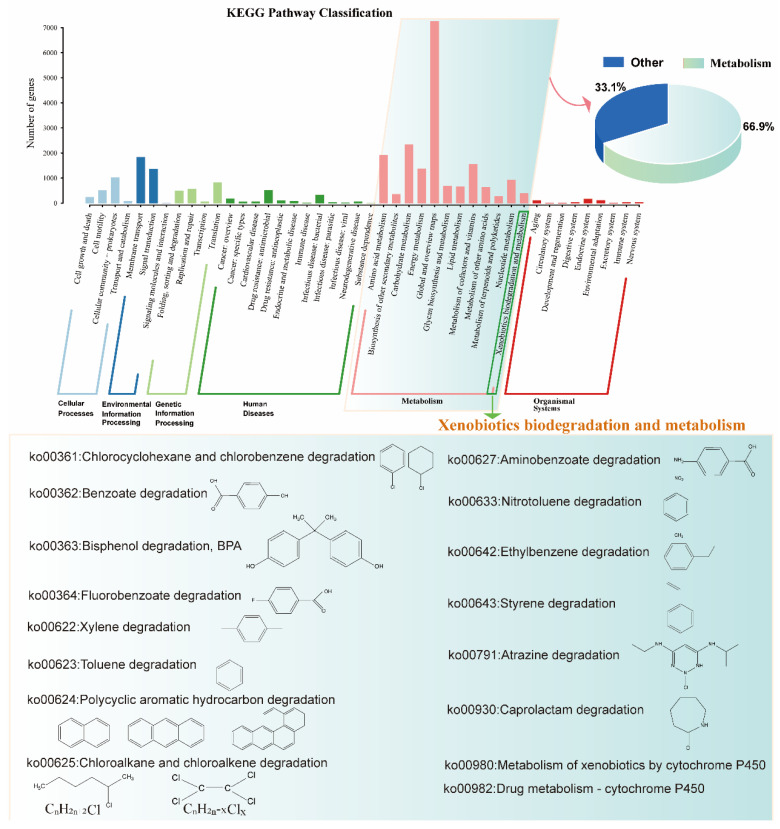
KEGG-based functional classification and xenobiotic degradation potential of the gut microbiota of *T. molitor*.

In this study, KEGG annotation and pathway analysis of the NE-01 genome were performed. Metabolism-related genes predominated (approximately 66.9%), among which xenobiotics biodegradation and metabolism was particularly prominent. The functional genes of the gut microbiota were mainly assigned to metabolic pathways, with additional categories including cellular processes, environmental information processing, and genetic information processing (Figure 4). Within the metabolic pathways, xenobiotics biodegradation and metabolism accounted for a substantial proportion, indicating that the gut microbiota possesses significant potential for exogenous pollutant degradation. Specifically, annotated functional genes were involved in the degradation pathways of various aromatic and halogenated pollutants, including benzoate (ko00362), toluene (ko00623), xylene (ko00622), ethylbenzene (ko00642), styrene (ko00643), aminobenzoate (ko00627), nitrotoluene (ko00633), caprolactam (ko00930), and atrazine (ko00791). In addition, pathways associated with the degradation of chlorocyclohexane/chlorobenzene (ko00361), bisphenol A (ko00363), fluorobenzoate (ko00364), polycyclic aromatic hydrocarbons (ko00624), and chloroalkane/chloroalkene (ko00625) were also detected, reflecting the broad-spectrum metabolic potential of NE-01 toward diverse environmental pollutants. Further analysis revealed that multiple xenobiotic degradation pathways were closely associated with cytochrome P450-related modules, including drug metabolism (ko00982) and metabolism of xenobiotics (ko00980). This suggests that gut bacteria may initiate the degradation of diverse pollutants through P450-mediated oxidation reactions. Such enzymes can introduce hydroxyl groups or cleave C–C/C–halogen bonds, thereby converting recalcitrant compounds into intermediates more amenable to further metabolism. The cytochrome P450-related pathways (ko00980, ko00982) were significantly detected, indicating that these microbial communities can mediate oxidation, hydroxylation, and detoxification reactions via P450 enzymes to achieve the initial activation of complex xenobiotics, laying the foundation for subsequent metabolism and energy conversion.

### 3.5. Putative Metabolic Pathways of PE, PET, PS, and DEHP in the Gut Microbiota of T. molitor

**Figure 5:**
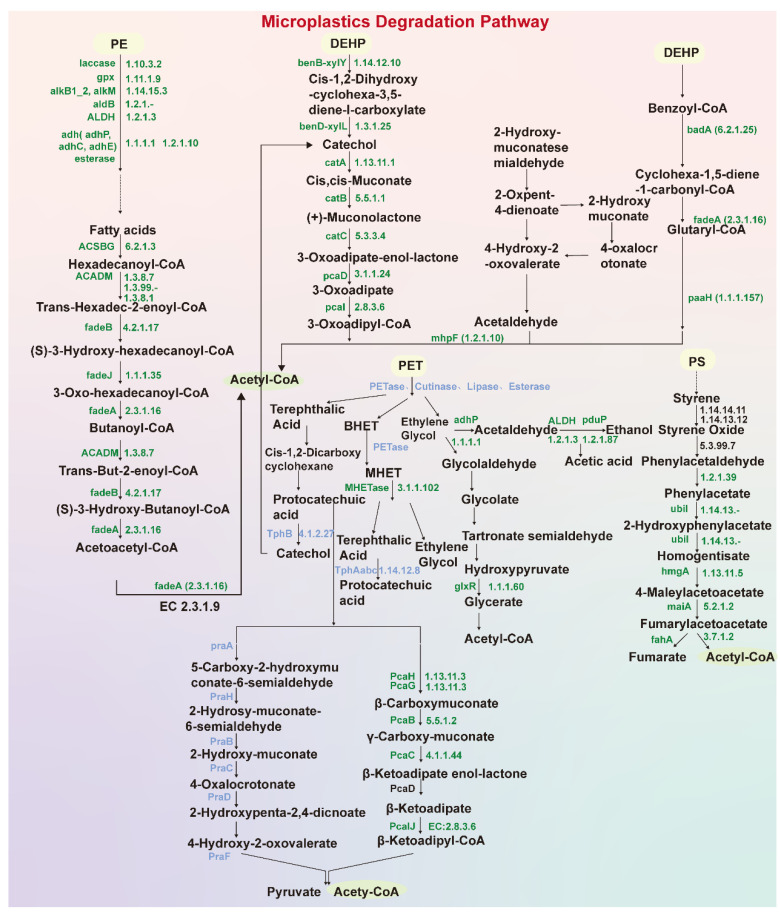
Summarizes the putative degradation pathways based on gene-encoded enzymes identified in the metagenome (This figure illustrates the potential enzymatic steps involved in the degradation of PS, PE, and PET by the NE-01 consortium. Enzymes directly annotated in the metagenome are highlighted in green, whereas enzymes inferred based on low sequence similarity (10–30% identity) are shown in blue. The arrows indicate the sequential conversion of polymer fragments into smaller intermediates and final metabolic products).

Metagenomic annotation of NE-01 revealed multiple functional genes and pathways associated with the degradation of typical plastics (PS, PE, and PET) as well as DEHP. Metagenomic annotation of NE-01 revealed multiple genes encoding putative enzymes and pathways potentially involved in the degradation of typical plastics (PS, PE, and PET) as well as DEHP. Based on gene annotation and sequence alignment, the overall results are summarized in Figure 5 (with enzymes directly annotated shown in green, and those identified by sequence similarity in other colors).

In the metagenomic annotation of this study, two previously reported microbial pathways for DEHP degradation [30] were successfully identified in the gut microbiota of *T. molitor*. One is the benzoate degradation pathway, in which genes such as *benB-xyiY* (EC 1.14.12.10) and *benD-xyiL* (EC 1.3.1.25) gradually convert substrates into catechol, which is subsequently cleaved by *catA* (EC 1.13.11.1), *catB* (EC 5.5.1.1), and *catC* (EC 5.3.3.4), entering the β-ketoadipate pathway through ortho- or meta-cleavage and ultimately producing 3-oxoadipyl-CoA [31]. The other is the benzoyl-CoA pathway, starting with *badA* (EC 6.2.1.25) catalyzing the formation of benzoyl-CoA, followed by the actions of *fadeA* (EC 2.3.1.16) and *paaH* (EC 1.1.1.157) to generate glutaryl-CoA, which is further funneled into central metabolism. In addition, some intermediates, such as acetaldehyde, could be converted into carbon metabolism via *mhpF* (EC 1.2.1.10).Overall, these pathways converge at acetyl-CoA as a key product. This metabolite is not only the central intermediate in DEHP degradation but also a common node in the degradation of PS, PE, and PET microplastics, enabling further entry into the tricarboxylic acid (TCA) cycle, acetate/dicarboxylate metabolism, or butanoate metabolism, and eventually complete mineralization into CO_2_ and H_2_O.

In the PE degradation pathway, the degradation of PE mainly proceeds through three stages: oxidative chain scission, fatty acid activation, and β-oxidation. In the initial stage, laccase (EC 1.10.3.2), gpx (EC 1.11.1.9), *alkB* monooxygenase (EC 1.14.15.3), alcohol dehydrogenase (EC 1.1.1.1/1.2.1.10), and aldehyde dehydrogenase (EC 1.2.1.3) catalyze the stepwise oxidation and cleavage of PE molecular chains, generating low-molecular-weight fatty acids. Subsequently, ACSBG (EC 6.2.1.3) activates fatty acids into acyl-CoA. During the β-oxidation cycle, ACADM (EC 1.3.8.7), *fadeB* (EC 4.2.1.17), *fadeJ* (EC 1.1.1.35), and *fadeA* (EC 2.3.1.16) catalyze the sequential reactions of dehydrogenation, hydration, further dehydrogenation, and thiolysis of acyl-CoA, progressively producing short-chain acyl-CoAs and ultimately acetyl-CoA, which enters the tricarboxylic acid (TCA) cycle for metabolic utilization. This indicates that the consortium is capable of degrading and assimilating alkane-based plastics via fatty acid metabolic pathways.

PS can be depolymerized into styrene, toluene, xylene, and other intermediates under the action of a series of depolymerases, some of which have been detected as products in PS-degrading strains. Putative pathway genes related to styrene degradation were annotated. The degradation of PS begins with monooxygenases (EC 1.14.14.11/1.14.13.12) converting styrene into styrene oxide or phenylethanol, which is further transformed into phenylacetaldehyde by oxidoreductases (EC 5.3.99.7). Phenylacetaldehyde is subsequently oxidized to phenylacetate by aldehyde dehydrogenase (EC 1.2.1.39), and then converted into 2-hydroxyphenylacetate by UbiI (EC 1.14.13.-). The products then enter the homogentisate pathway, where *HmgA* (EC 1.13.11.5) catalyzes the formation of 4-maleylacetoacetate, which is further metabolized by *MaiA* (EC 5.2.1.2) and *FahA* (EC 3.7.1.2) into fumarate and acetyl-CoA, respectively, ultimately feeding into the TCA cycle.

In the polyethylene terephthalate (PET) degradation pathway, PET is initially hydrolyzed by hydrolases such as PETase, cutinase, lipase, and esterase, which cleave ester bonds to produce terephthalic acid (TPA), bis(2-hydroxyethyl) terephthalate (BHET), and mono(2-hydroxyethyl) terephthalate (MHET). Among these, MHET is further degraded by MHETase (EC 3.1.1.102) into TPA and ethylene glycol (EG). TPA can be catalyzed by *TphB* (EC 4.1.2.27) and *TphAabc* (EC 1.14.12.8) into catechol and protocatechuic acid, which subsequently enter the aromatic compound metabolic pathways. On the other hand, EG is sequentially oxidized by *adhP* (EC 1.1.1.1) and ALDH (EC 1.2.1.3/1.2.1.87) into acetaldehyde, acetate, and acetyl-CoA. Its metabolic intermediate glycolaldehyde can also be converted by *glxR* (EC 1.1.1.60) into hydroxypyruvate and glycerate, which ultimately generate acetyl-CoA and feed into the tricarboxylic acid (TCA) cycle.

## 4. Discussion

Studies on microbial community-mediated degradation of microplastics remain limited, and the mechanisms involved as well as the participation of plastic additives require further exploration. *Pseudomonas* has been reported as an efficient degrader of DEHP [11] and also plays an important role in plastic degradation [32]. *Morganella*, *Hafnia*, *Klebsiella*, *Escherichia*, and *Salmonella* have previously been considered major contributors to plastic degradation [33,34,35], and all were detected in the gut-derived microbial consortium NE-01 in this study, further confirming its potential for the degradation of diverse microplastics.

For typical microplastics that contain only C–C bonds without oxygen atoms, such as PS and PE, their microbial degradation generally involves a primary oxidation stage and a secondary metabolic stage. In the primary oxidation stage, bacteria colonize the plastic surface through secretion of extracellular enzymes and the formation of biofilms, which promote surface erosion and increase hydrophilicity. Key enzymes such as laccases, peroxidases, and oxygenases first catalyze long-chain polymers into fatty acids or oligomers [36]. The active site of laccases contains four copper ions; PE can directly react with the T1 copper ion, releasing electrons to form PE radicals, which then undergo cleavage into alkyl radicals that are further oxidized into alcohols, aldehydes, and carboxylic acids, and finally mineralized into CO_2_ and H_2_O [33,37]. *AlkB* alkane hydroxylases have also been demonstrated to participate in PE degradation [38]. In the secondary metabolic stage, primary oxidation products such as oligomers and small molecules are transported into cells and converted into short-chain fatty acids or organic acids through the actions of oxygenases, dehydrogenases, esterases, lipases, hydrolases, and cytochrome P450 enzymes [38,39]. Lipases assist in the oxidative degradation of PE, while alcohol dehydrogenases (adhE) and aldehyde dehydrogenases (ALDH family) oxidize fatty alcohols and aldehydes into short-chain fatty acids, which then enter central metabolic pathways. Glutathione peroxidase [39] and other peroxidases promote polymer oxidation through redox reactions [26], while esterases (*frsA*) and carboxylesterases (*tesB*) enhance microbial adhesion and degradation efficiency through depolymerization and surface modification. The synergistic action of these enzymes enables PE to be broken down into low-molecular-weight compounds such as alkanes, alkenes, ketones, aldehydes, alcohols, and fatty acids. For PS, depolymerization occurs under radical attack, generating styrene, toluene, xylene, and their derivatives that contain aromatic rings [40]. PS degradation involves β-carbon attack on the polymer backbone as well as cleavage of the aromatic side chains [41], thereby driving the stepwise transformation of PS into smaller and more metabolically accessible intermediates [41]. The oxidation of the styrene side chain involves the styrene monooxygenase system composed of *StyA* and *StyB*, together with styrene oxide isomerase and phenylacetaldehyde dehydrogenase [42,43]. The combined action of these enzymatic cascades not only enables the conversion of styrene into phenylacetic acid but also facilitates the smooth channeling of metabolic flux into central metabolic pathways. Phenylacetaldehyde dehydrogenase exhibits functional versatility, as it is not only involved in styrene side-chain oxidation [43,44] but also plays a role in the metabolism of phenylethanol, toluene, ethylbenzene, and other aromatic compounds [45].

At present, most highly efficient PET-degrading enzymes have been isolated from plastic-polluted environments such as landfills and marine ecosystems, while reports of related enzymes derived from animal sources remain scarce [46]. Yoshida et al. reported in Science that the newly discovered bacterium *Ideonella sakaiensis* 201-F6 secretes a PET hydrolase, *Is*PETase, which enables the effective degradation of PET [47]. When growing with PET as the sole carbon source, this strain secretes two enzymes that hydrolyze PET, producing mono(2-hydroxyethyl) terephthalate (MHET) as the main intermediate, with *Is*PETase serving as the key enzyme catalyzing this reaction. Both PETase and MHETase belong to the α/β-hydrolase superfamily [48]. Known PET hydrolases include cutinases [49], lipases, esterases, and PETase [50,51,52,53], whose catalytic activity relies on the catalytic triad composed of serine (Ser), histidine (His), and aspartic acid (Asp), with serine acting as the nucleophile at the core of the hydrolytic reaction [54]. PETase hydrolyzes PET into ethylene glycol (EG) and terephthalic acid (TPA) through a two-step acylation–deacylation process, providing the basis for PET recycling and upcycling [52,55,56,57]. In this study, the gut-derived microbial consortium NE-01 also exhibited significant PET degradation capability (a weight loss rate of 16%). MHETase (EC 3.1.1.102) was annotated in NE-01, suggesting that animal gut microbiota may harbor PET hydrolases or related metabolic pathways, thereby providing novel biological resources and potential application value for PET biodegradation.

The major pollution sources of microplastics (MPs) include both the polymer particles themselves and the plasticizers they contain, but the microbial degradation pathways of the two differ substantially. Microorganisms play a key role in the decomposition and mineralization of DEHP through enzyme-driven processes controlled by specific functional genes. Previous studies have reported that in the DEHP degradation pathway, key genes of both aerobic and anaerobic routes were detected in earthworm manure compost [30]. These genes are involved in the transformation of phthalates and their metabolic intermediates, ultimately generating acetyl-CoA and succinyl-CoA via the β-ketoadipate pathway, indicating that microbial communities are able to degrade synthetic plasticizers through pathways analogous to natural aromatic compound metabolism.

In summary, although the gut microbiota of *T. molitor* is known to possess plastic-degrading potential, its activity typically depends on the physiological state and living environment of the host insect. To overcome this limitation and explore its biotechnological applications, we enriched the gut-derived microbial community under in vitro conditions. The results showed that the enriched consortium retained measurable plastic-degrading activity outside the host, indicating that insect gut microorganisms can maintain their degradative potential even after being separated from the host environment. It should be emphasized that not all members of the enriched community are capable of directly degrading plastics. However, the overall degradation performance reflects synergistic interactions among different microbial taxa. Previous studies have shown that non-degrading bacteria can enhance the performance of degraders through nutrient exchange, detoxification, or co-metabolic processes, thereby improving overall degradation efficiency [58]. From this perspective, the purpose of this study was not to isolate single plastic-degrading strains or to investigate community shifts using plastics as the sole carbon source, but rather to determine whether a gut-derived microbial consortium could still exhibit community-level degradation potential after being decoupled from the host. This enrichment-based strategy provides a new approach to “de-host” insect gut microbiota while maintaining their ecological interactions and enabling controllable in vitro applications. It not only demonstrates the feasibility of rapidly enriching microorganisms with plastic-degrading potential, but also reveals, at the genomic level, the presence of multiple plastic-degrading genes within the consortium. Through metagenomic annotation, we identified several metabolic pathways involved in the degradation of PE, PET, PS, and the plasticizer DEHP, all of which correspond to the core enzymes and key metabolic nodes previously reported in microbial plastic-degradation studies. Given that the main objective of this study was to preliminarily explore, from a genomic perspective, the potential plastic-degrading capability of the gut-derived microbial consortium, we inferred the possible occurrence of biodegradation primarily based on the consistency between the observed film weight loss and the presence of key degradation-related genes and metabolic pathways identified by metagenomic analysis. However, we also acknowledge that weight loss alone is insufficient to fully confirm microbial utilization of plastics, and that this study did not directly detect soluble degradation intermediates. Future research should integrate time-resolved gas chromatography–mass spectrometry (GC–MS) or liquid chromatography–mass spectrometry (LC–MS) analysis of degradation products to more explicitly link functional genes with actual metabolic outputs, thereby enabling a more accurate elucidation of microbial plastic degradation mechanisms. These findings provide an important theoretical foundation for developing microplastic bioremediation strategies based on insect gut microbiota.

## 5. Conclusions

This study systematically evaluated the potential of the cultivable gut-derived microbial consortium NE-01 from *T. molitor* in degrading multiple types of plastics and plasticizers. The results demonstrated that NE-01 was capable of effectively degrading common microplastics, including PS, PE, and PET. Metagenomic analysis further revealed the presence of multiple metabolic pathways and key functional genes associated with plastic degradation, encompassing several putative degradation routes for PS, PE, and PET, as well as both aerobic and anaerobic pathways for the degradation of the plasticizer DEHP. This study not only confirms the important role of insect gut-derived microbial communities in plastic biodegradation from the perspectives of microbial ecology and metabolic potential, but also provides essential theoretical support and promising application prospects for the large-scale enrichment of plastic-degrading microbial consortia and the development of microbe-based plastic degradation formulations in the future.

## Data Availability

The original contributions presented in this study are included in the article. Further inquiries can be directed to the corresponding author.
